# Eukaryotic Ribosomal Protein S5 of the 40S Subunit: Structure and Function

**DOI:** 10.3390/ijms24043386

**Published:** 2023-02-08

**Authors:** Lijuan Qiu, Wen Chao, Shan Zhong, An-Jing Ren

**Affiliations:** Experimental Teaching Center, College of Basic Medical Sciences, Naval Medical University, Shanghai 200433, China

**Keywords:** ribosomal protein S5, translation, liver disease, cancer, function

## Abstract

The ribosomal protein RPS5 is one of the prime proteins to combine with RNA and belongs to the conserved ribosomal protein family. It plays a substantial role in the process of translation and also has some non-ribosome functions. Despite the enormous studies on the relationship between the structure and function of prokaryotic RPS7, the structure and molecular details of the mechanism of eukaryotic RPS5 remain largely unexplored. This article focuses on the structure of RPS5 and its role in cells and diseases, especially the binding to 18S rRNA. The role of RPS5 in translation initiation and its potential use as targets for liver disease and cancer are discussed.

## 1. Introduction

The ribosome is the important site of protein synthesis in all kingdoms of life [1]. Ribosomes are cellular ribonucleoprotein particles, mainly composed of rRNA and proteins. Its basic function is to synthesize protein polypeptide chains by amino acids according to the instructions of mRNA. The ribosome comprises a large subunit (L) and a small subunit (S), each of which consists of rRNAs and ribosomal proteins (RPS). Many ribosomal proteins are conserved across all kingdoms [2]. For example, of the 79 ribosomal proteins in the yeast ribosome, 47 have homologous archaeal and bacterial counterparts [3,4]. Previous studies have shown that ribosomal proteins play important roles in ribosome assembly and protein translation, but other biological functions remain ill-defined [5]. Ribosomal proteins are involved in the regulation of protein synthesis and play distinct roles in the process of translation. There are more than 80 kinds of ribosomal proteins (ribosomal protein, RP) found in eukaryotic cells, which are widely distributed in various tissues. The designation of ribosomal proteins is related to the proteins of the large and small subunits of the ribosome. The small subunit ribosomal proteins were named S1–S31, and the large subunit ribosomal proteins were named L1–L44.

Eukaryotic ribosomal protein S5 (RPS5) is located at the head of the 40S ribosomal subunit and is a member of a highly conserved ribosomal protein family. RPS5 is a basic protein containing many arginyl and lysyl residues, with an estimated pI of 9.6, and is essential for cell viability [6,7,8,9,10]. Most studies believe that RPS5 is very characteristic in amino acid composition because it contains a large amount of arginine and lysine, it is alkaline, and the basic amino acids are more concentrated [7,9]. RPS5 forms part of the exit (E) site and cross-links with the E-site tRNA [11,12]. In vivo depletion of RPS5 leads to the nuclear entrapment and degradation of nascent SSUs and a delay of early nuclear pre-18S rRNA processing events [13,14]. RPS5 can combine with the initiation factors eIF3 and eEF1A or interact with eIF2αto affect the efficiency of initiation [13,15,16,17]. In addition to its important role in the process of translation, RPS5 has significant non-ribosomal functions. For example, RPS5 is associated with cell division and apoptosis and is involved in the development of liver hepatitis and fibrosis [18,19,20]. Circ-RPS5-modified ADSC exosome improved cognitive function by decreasing neuronal damage and shifting microglia from an M1 to M2 phenotype in the hippocampus [21,22,23]. In recent years, increasing evidence has shown that many ribosomal proteins, in addition to their constituent ribosome and involved in protein biosynthesis, are also associated with certain diseases. For example, it was recently found that the RPS19 gene has a mutation in congenital aplastic anemia and that RPL 6 is associated with congenital heart disease. In mammals and *Drosophila*, RPS3 has an endonuclease action, while *Drosophila* RPS6 has a tumor-suppressing effect, and human RPL5 can form the protein complex with Mdm 2 and p53.

In this review, we discuss the current research on the structure and function of eukaryotic RPS5, the relationship between structure and function in the N-terminus in the process of translation initiation, and in particular, advances in medical research.

## 2. The Structure of RPS5

Scanning RPS5 genes from distinct eukaryotic species revealed that the rat and yeast RPS5 genomes are represented by a single gene copy, whereas mice and humans have three to six copies [8,9,10]. The human RPS5 gene is comprised of 701 nucleotides and encodes 204 amino acids. There are 37 bases in the 5′-end noncoding region, 612 bases linked to the coding region, and a 51 bases noncoding sequence linked to Poly (A) in the 3′-end region [9,23]. Interestingly, the single rat RPS5 gene similarly encodes 204 amino acids [9]. Furthermore, the amino acid sequences of rat RPS5 and human RPS5 are 98% identical, with only three amino acid differences: T6, G60, and R181 in the rat protein are substituted by A6, A60, and A181 in the human protein [9,10]. Eukaryotic RPS5 amino acid sequences contain more basic amino acids than acidic amino acids. Interestingly, these basic and acidic amino acids, particularly the N-terminal and C-terminal charged amino acids, tend to form several substantially hydrophilic and hydrophobic regions, respectively [23]. Although the amino acid sequences of eukaryotic RPS5s differ, they share a highly conserved C-terminal structure and variable N-terminal structure (Table 1) [22]. Both RPS5 and RPS5a are transcripts derived from the same cDNA, and RPS5a is a breakdown product of RPS5, except for RPS5, which is not at the amino terminus five residues, and both have the same amino acid sequence. Whether part of the decomposition transition of RPS5 to RPS5a is physiological or accidental is currently unknown [7].

In prokaryotes, ribosomal protein S7 is homologous to eukaryotic RPS5 and shares approximately 30% identity at the amino acid level, but the function of RPS5 is more complex than that of RPS7 during translation [7,9,11,24,25]. A comparison of the amino acid sequences of rat RPS5 and proteins from 17 prokaryotic species revealed 16 conserved amino acids in the C-terminus, particularly three alanines [7].

**Figure 1:**
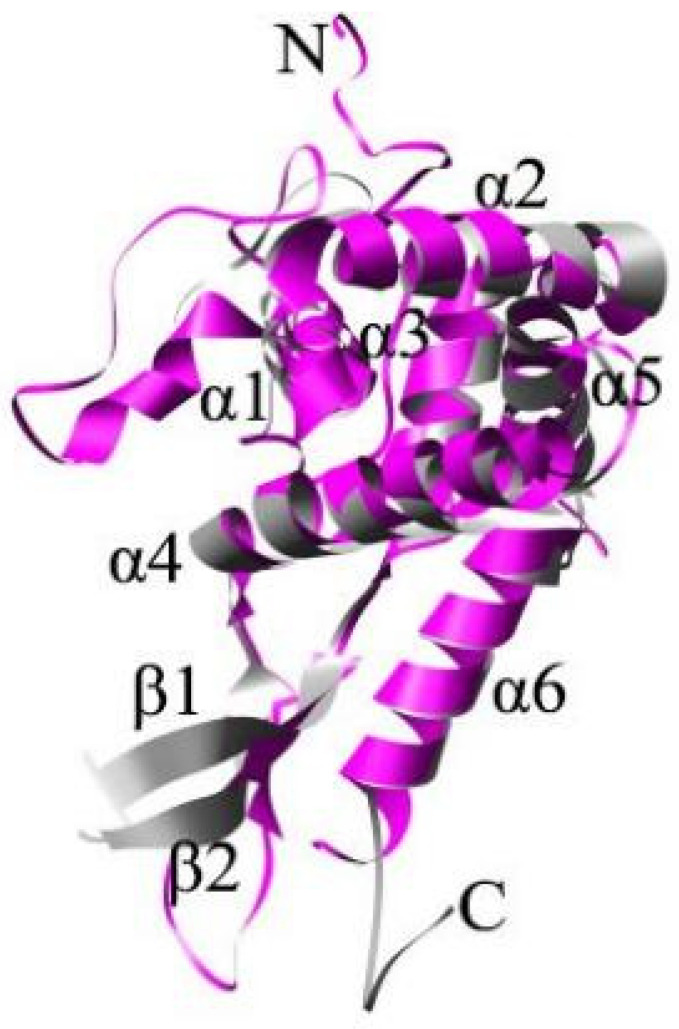
Three-dimensional structure of RPS5 has a common backbone comprising six α-helices, two β-strands, and seven loops.

Many three-dimensional structures of prokaryotic ribosomal protein S7 have been revealed by X-ray crystallography. These structures show that RPS7 consists of a short 310 helix, two β-strands, and six α-helices, which form a hydrophobic stable α core and a β-arm, with seven loops connecting these α-helices and β-strands [25,26,27]. In addition, some basic amino acids and hydrophobic residues form a concavity on the surface of RPS7 [28,29,30,31]. However, in contrast to prokaryotic RPS7, no crystal data have been reported for eukaryotic RPS5 proteins; although the 80S and 40S ribosomes have been successful crystal 80S and 40S structures, the three-dimensional structure of RPS5 has been isolated and compared with that of prokaryotic RPS7. RPS5 and RPS7 have a common backbone comprising six α-helices, two β-strands, and seven loops. However, eukaryotic RPS5 has an N-terminal extension of approximately 60 residues that forms an irregular curl and a C-terminal truncation of approximately seven residues [27,32] (Figure 1).

## 3. Role of RPS5 in Translation

In prokaryotes, RPS7 plays a central role in the progress of translation [25,26,28,33]. RPS7 is the primary protein located on the head of the 30S subunit and binds to the lower half of the 3′ major domain of the 16S rRNA, which consists of helices H28-30 and H38–H43(926–986/1219–1393) [33,34,35,36]. It initiates the assembly and folding of the 30S ribosomal subunit [37]. In addition, RPS7 can cross-link to the P and E sites of tRNA and act as a translational feedback repressor, binding not only upstream of the decoding region of mRNA but also to the str operon of mRNA [33,37,38,39]. Based on research on the interaction of RPS7 and RPS11 in *E. coli*, mutations of RPS7 can affect translational fidelity, leading to an increase in the probability of frameshifting and codon misreading [40].

Eukaryotic RPS5, like its prokaryotic counterpart RPS7, can also cross-link to the 18S rRNA and to position-3 of the derivatized mRNA [32]. In addition, RPS5 participates in the progress of protein synthesis, is involved in the regulation of cell differentiation and apoptosis, and interacts with other ribosomal proteins on the surface of 40S to contribute to the translation function of the ribosome [19,41,42]. The human 18S rRNA region interacts with RPS5, and the binding fragment on the 18S rRNA is helices H28–H30 and H41–H43 (1203–1236/1521–1698), which is homologous to the prokaryotic RPS7-16S rRNA binding fragment H28–H30 and H38–H43 (926–986/1219–1393) [41]. Further studies demonstrate that the interaction of RPS5 and RPS16 modulates the binding of the C-terminus of RPS16 to Met-tRNAi Met to promote a functional 48S preinitiation complex (PIC) in the process of yeast translation [15]. Interestingly, human RPS5 and RPS16 can bind to the same 18S rRNA region, H28–H30 and H41–H43; this interaction is essential for translation, and the binding of RPS5 has an apparent synergetic effect of protecting the site from hydrolysis by RNases when RPS5 and RNases simultaneously bind to 18S rRNA [15,41,42]. The TATA-box binding protein (TBP) is a subunit of the multi-protein TFIID complex believed to be critical in this process. Le reconstituted transcription from highly purified components on a ribosomal protein gene (RPS5) and discovered that TFIIDΔTBP binds and rearranges the promoter DNA topology independent of TBP [43]. TFIIDΔTBP binds the ~200 bp promoter and changes the DNA topology to a larger extent than the nucleosome core particle. TBP inhibited the DNA binding activities of TFIIDΔTBP, and the complete TFIID complex may represent an auto-inhibited state. Furthermore, the DNA binding activities of TFIIDΔTBP were necessary for the assembly of a PIC poised to select the correct transcription start site (TSS).

## 4. Potential rRNA Binding Sites on the N-Terminus of RPS5

**Figure 2:**
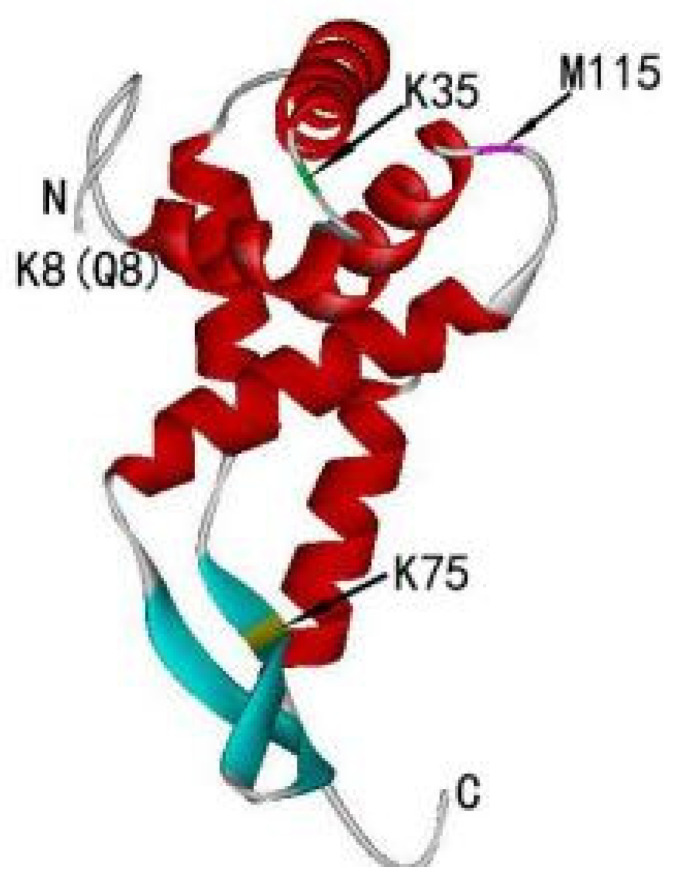
Ribbon representation of the RPS7 protein structure (PDB:1RSS). The red domain is M115, 146 the green domain is K35, and the yellow domain is K75. Regions deleted by mutagenesis are shown in 147 aqua, but residues P1-G11 are absent in this structure.

The structural data suggest that the N-terminus of prokaryotic RPS7 plays an extremely critical role in the progress of protein synthesis. The N-terminus also cross-links to an antibiotic that binds to the A site and is involved in the binding of puromycin [44]. The close contact of RPS7 with ribosomal RNA contributes to the progress of translation [27,33]. The binding sites of RPS7 on 16S rRNA are Q8, K75, M115 in *E. coli* S7 and K8 and M115 in BstS7 [45,46,47,48]. Q8 (K8) and K75 are both cross-linked to C1378 of 16S RNA, which is part of the P site, and M115 is cross-linked to U1240. In addition, F17, K35, G54, and K113 all affect binding [49]. These residues belong to the concave domain of the protein, and most of these residues are also in the N-terminal region [38]. K35 is required for the efficient assembly of S7 into 30S subunits in vivo and may directly contact the mRNA. Truncating the N-terminus of *E. coli* RPS7 decreases the affinity of RPS7 for 16S rRNA [36] (Figure 2).

**Figure 3:**
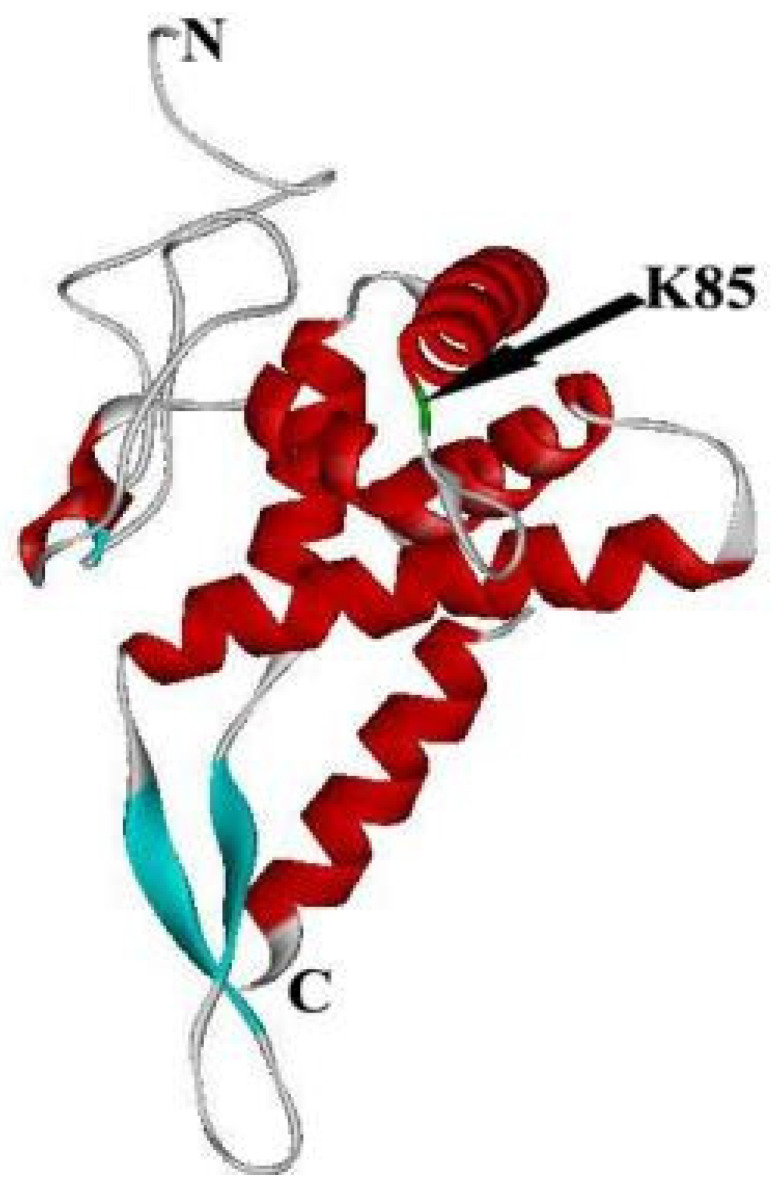
Crystal structure of Homo RPS5 is separated from the structure of Homo 40S using software 167 of chimera. The green domain is K85.

Similar to prokaryotic RPS7, the N-terminus of eukaryotic RPS5 also plays a significant role in the translation process, particularly in translational initiation [17]. The binding sites of RPS5 on 18S rRNA correspond to the RPS7-16S rRNA binding fragment region [30,41]. By overexpression of RPS5 and knockdown of cells from different sources, RPS5 interacted with viral RNA terminal sequences, which is a non-structural protein of the virus [41]. The difference in RPS5 binding ribosomal protein before and after viral infection elucidates the molecular mechanism of RPS5 promoting viral protein translation at the cellular level [50]. RPS5 and eIF2a are in close proximity in the PIC, and the N-terminus of RPS5 is cross-linked to eIF2α to maintain the correct positioning of eIF2a relative to other PIC components and ensure start-codon selection by the PIC [16,17]. The fragments 72–85 and 2–18 of RPS5 cross-link with fragments 81–87 and 68–75 of eIF2a, respectively [51]. These RPS5 fragments are both located at the N-terminus; fragment 72–85 is located in the conserved region, while fragment 81–87 is located in the N-terminal extension [32]. As mentioned previously, K35 of prokaryotic RPS7 is one of the binding sites that affect the progress of translation. K85 of human and yeast RPS5 is homologous to E. coli RPS7 K35, suggesting that eukaryotic RPS5 Lys85 cross-links with 18S rRNA. Interestingly, Lys85 is a highly conserved amino acid residue among eukaryotic species (Figure 3).

**Figure 4:**
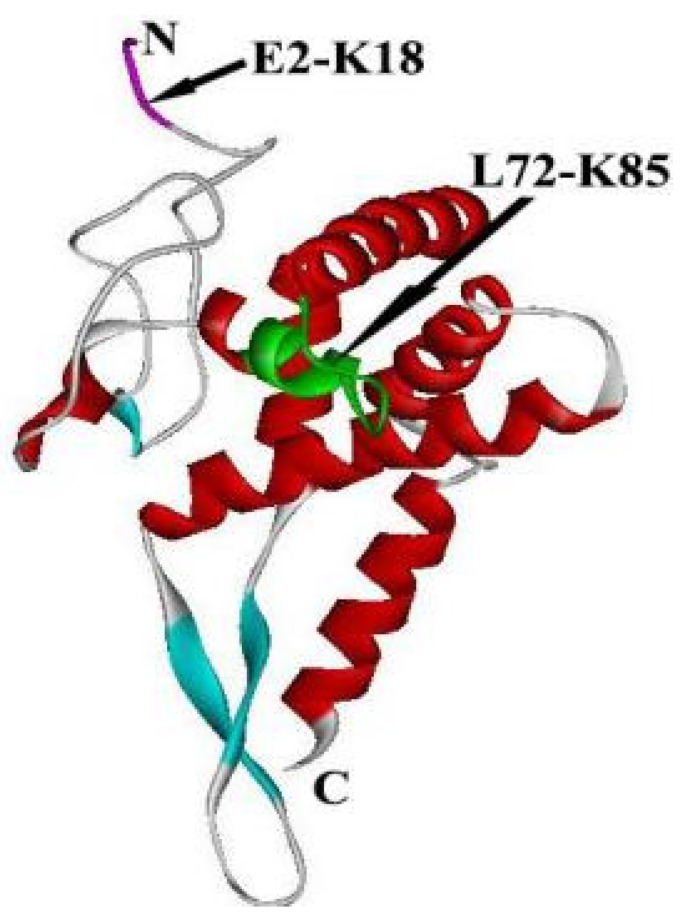
Crystal structure of Homo RPS5 is separated from the structure of Homo 40S using software of chimera. The red fragment is T2-K18, and the green fragment is L72-K85. Regions deleted by mutagenesis are shown in aqua, but residues M1-E13 are absent in this structure.

Because the translation system is more complex in eukaryotes than in prokaryotes, the eukaryotic RPS5-18S rRNA interaction may include additional binding sites that are in- consistent with RPS7-16S rRNA interactions [30,41]. Cryo-EM data indicate that the eukaryotic 40S subunit has an extension region that is involved in the RPS5-18S rRNA interaction compared with the 30S subunit [52]. This extension region may be formed by the portion of the RPS5 N-terminus that cross-links with several regions of 18S rRNA. Mutation of the yeast RPS5 N-terminus adversely affected translational efficiency and fidelity, as well as the formation of 48S PIC [13,17]. Replacement of yeast RPS5 with human RPS5 lacking 21 amino acid residues and truncation of 46 amino acid residues of yeast RPS5 affect the elongation factors, increase frameshifting for translation, and reduce the efficiency of UAA stop codon recognition [13,22]. Surprisingly, the RPS5 core promoter can be activated by various activation domains fused to a GAL4 DNA binding domain but not by the original upstream activating sequence (UAS) of the RPS5 gene. These results confirmed that there is a second binding site in the N-terminal extension of RPS5, possibly located at fragments 2–18 (Figure 4).

**Figure 5:**
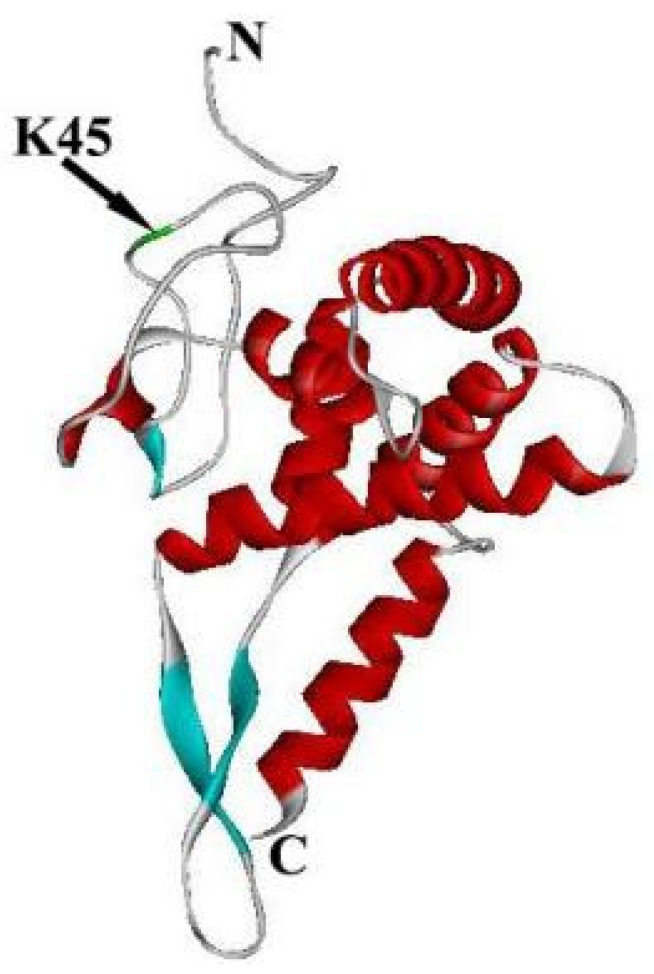
Crystal structure of Homo RPS5 is separated from the structure of Homo 40S using software of chimera. The green domain is K45.

In addition, the cross-links of the N-terminus of RPS5 to the N-terminus of RPS16 regulate the interaction of the C-terminus of RPS16 with Met-tRNAi Met to maintain the accuracy of the PIC function [15,53,54]. The crystal structure of 80S showed that K45 in the N-terminus of RPS5 is close to F46 of RPS16 [15,30]. Moreover, K45 is also one of several highly conserved amino acids in mammals and fungi. Therefore, K45 might be the other potential binding site on the N-terminus of RPS5 that affects the translational process [15] (Figure 5).

The C-terminus of PRS5 may have a different function in the process of initiating protein synthesis [36,37,44,54]. The C-terminus of PRS5 is specifically required for the efficient final 3′ end maturation of 18S rRNA precursors [32]. Substitutions in β-strand-1 and C-terminal residues of yeast RPS5 reduce bulk initiation, confer ‘leaky-scanning’ of AUGs, and reduce initiation fidelity by exacerbating the effect of poor context of the eIF1 AUG codon to reduce eIF1 abundance [55]. It is crucial for both efficiently and accurately recognizing the start codon to begin translation.

## 5. RPS5 as a Potential Target for Liver Disease and Cancer

Although RPS5 is an important ribosomal protein in the 40S subunit involved in translation, it has many other non-ribosomal functions [14,56]. For instance, a previous study has proved that ribosomal protein S5 (RPS5) is a direct target of M19. During osteoclastogenesis, the RPS5 level in RAW264.7 cells was significantly down-regulated. In summary, RPS5 may be a key protein in PMOP [57].

**Figure 6:**
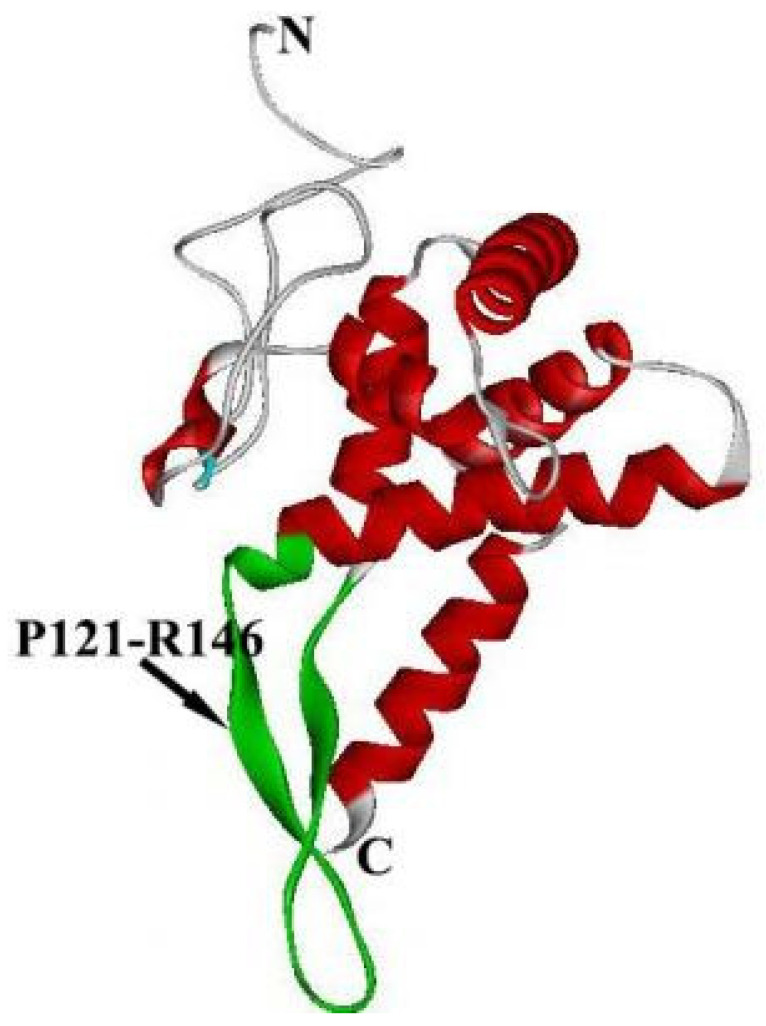
Crystal structure of Homo RPS5 is separated from the structure of Homo 40S using software of chimera. The green fragment is P121-R146.

RPS5 plays a unique role in some diseases. In the process of hepatitis C virus (HCV) translational initiation, the 5′ untranslated region (UTR) of the mRNA, which has no cap, is replaced by an internal ribosomal entry site (IRES) [58]. The structures of IRES-containing domains II, III, and IV are similar to that of tRNA [59]. The interaction of IRES with the 40S ribosomal unit initiates ribosomal translation of the viral mRNA [56,57,58,59,60,61,62]. Although the IRES does not bind directly to the 40S subunit, RPS5 is involved in the IRES-ribosomal interaction [55]. As a unique binding protein, RPS5 simultaneously binds to the IRES and 40S ribosomal unit [63]. The cryo-EM structure of the IRES-40S complex shows that RPS5 is close to region II of the IRES and has two binding fragments, P121-R146 and N179-R204, at the helix of the C-terminus, which cross-link to the region II fragments 70–74 and 92–97 of IRES, respectively, following the interaction between the AUG start codon and 18S rRNA in 40S [64,65]. The interaction causes a rearrangement of the 18S rRNA structure in the vicinity of the universally conserved nucleotide G1639 to adjust its conformation to facilitate the tRNA and Met-tRNAMet combination [63]. Recent studies have revealed that RPS5 fragment P121-R146 forms a β-hairpin to bind domains II and IV of IRES, which regulate the internal initiation of translation [56,66]. The ribosomal protein RPS5 was shown to interact with RHDV 3′ Ex RNA directly by RNA- pulldown and confocal microscopy. To further investigate the role of RPS5 in RHDV replication, small interfering RNAs for RPS5 and RPS5 eukaryotic expression plasmids were used to change the expression level of RPS5 in RK-13 cells; the results showed that the RHDV replication and translation levels were positively correlated with the expression level of RPS5. It was also verified that RPS5 promoted RHDV replication by constructing RPS5 stable overexpression cell lines and RPS5 knockdown cell lines. In summary, it has been identified the focus on the discovery and characterization of disease resistance protein RPS5, and the interaction of RPS5 and RHDV 3′ Ex RNA region, played a role in virus replication [67]. These data suggest that RPS5 may be a potential target for treating HCV and that the fragment P121-R146 warrants special attention (Figure 6). In addition, RPS5 is clearly associated with the progression of hepatic fibrosis. RPS5 not only inhibits hepatic stellate cell (HSC) activation but also contributes to the dephosphorylation at S473 and T308 of Akt, which leads to the phosphorylation of many different targets regulating several cell functions [20,68,69,70]. Moreover, knockdown and restraint of RPS5 overexpression can increase the progression of hepatic fibrosis induced by dimethylnitrosamine (DMN) or bile duct ligation (BDL), which is associated with the modulation of Akt phosphorylation and HSC number in the fibrotic liver [20] for sequenced cDNA of 715 bp (RPS5) from a murine erythroleukemia (MEL) cDNA library. The cloned mouse cDNA showed a significant degree of structural homology at the DNA and protein levels with the human and rat genes known to encode the 40S ribosomal subunit of the RPS5 protein. Using 715-bp cDNA as a probe, the presence of an RNA transcript in the cytoplasm of MEL and human neuroectodermal RD/TE-671 cells. The steady-state accumulation level of this RNA transcript decreased upon induction of differentiation of both cell lines by treatment with DMSO and UDP-4, two structurally different inducers. Blockade of MEL cell differentiation by the inhibitor N6-methyladenosine could preserve the constitutive expression of the RPS5 gene. DNA methylation analysis at CCGG sites located at the RPS5 gene locus in both undifferentiated and differentiated MEL cells revealed that the suppression of the RPS5 gene during differentiation of MEL cells was not associated with methylation changes at these sites. Therefore, we conclude that there may be a disparate pattern of expression of the RPS5 gene in differentiating and apoptotic cells. These data can be valuable for understanding the role of ribosomal proteins during differentiation and cell death (apoptosis) of neoplastic cells, although there is no experimental evidence that the suppression of the RPS5 gene is mechanistically related to the induction of differentiation. It is likely to be considered as part of the differentiation process [71]. The erythrocyte differentiation of MEL cells is associated with transcriptional activation of the genes encoding ribosomal RNA and RPS5 and RPSL35a proteins, and differentiation of MEL cells is accompanied by a marked downregulation of RPS5. RPS5 is maintained when differentiation of MEL cells is blocked, while RPS5 is continuously expressed in apoptotic cells, thus inferring that the RPS5 gene is expressed differently in differentiated and apoptotic cells.

At both the early and the progression stages of the abdominal aortic aneurysm, RPS5 was identified as the hub gene, and the current study indicated that glycosaminoglycan degradation, anaerobic metabolism, and mitochondrial dysfunction played a critical role in the formation of abdominal aortic aneurysm. These data demonstrate that RPS5 may be a potential target for therapeutic intervention in hepatic fibrosis [72]. In addition to its involvement in fibrotic liver diseases, RPS5 also correlates with some cancers. Inhibition of RPS5 overexpression can prevent the development of colorectal cancer and nasopharyngeal cancer [8,73].

However, the relationship between RPS5 and the mechanism underlying the development of these diseases is unclear. RPS5 warrants further in-depth study as a promising potential target for the treatment of these diseases.

## 6. The Non-Ribosomal Functions of the RPS5 N-Terminus

In addition to its important role in regulating protein translation in cells, RPS5 has many non-ribosomal functions that have received widespread attention. A number of ribosomal proteins have been reported to regulate cell proliferation and induce cell death [19]. For example, two RPS5 genes, AtRPS5A and AtRPS5B, which encoded by *Arabidopsis*: Mutation of AtRPS5A, which is strongly expressed in dividing cells, causes semi-dominant developmental phenotypes. By contrast, AtRPS5B is strongly expressed during cell differentiation [74]. Current evidence suggests that RPS5 accumulation in MEL cells is correlated with the initiation of MEL cell differentiation and regulation of the levels of the cyclin-dependent kinases CDK2, CDK4, and CDK6 [18,19]. In addition, the deregulation of RPS5 gene expression affects cell entrance into G1/G0 phase cycle arrest and delays the onset of initiation of erythroid maturation [18,19]. RPS5 is not phosphorylated in the 40S ribosomal unit but is phosphorylated when present in its free form [68,75,76,77]. Phosphorylation of RPS5 promotes the protein’s movement from cellular compartments into nucleoli [57]. In MEL cells, phosphorylation of T133 on the C-terminus of RPS5 can trigger S24 phosphorylation on the N-terminus, which is essential for the entry of this protein into nucleoli. Furthermore, confocal microscopy revealed that RPS5 N-terminal region 1–50 is involved in the protein’s cellular traffic. The cellular level of RPS5 is regulated by proteolysis occurring within its N-terminal region, which harbors poor PEST structural elements, and this proteolytic attack is probably dependent on phosphorylation [57]. The cellular traffic and cellular levels of RPS5 are closely correlated with its N-terminus.

## 7. Conclusions

*Piroplasmosis* is a serious, debilitating, and sometimes fatal disease. The nuclear genes 40S RPS5 and the mitochondrial DNA Cytochrome c oxidase subunit III (cox3) gene were used to investigate their relative resolution in the phylogeny of isolates from *Babesia* and *Theileria*. The results showed that the combined DNA sequences of the nuclear RPS5 and cox3 genes improved the evolutionary relationships between *Babesia* and *Theileria* species. The mitochondrial cox3 gene outperforms the nuclear RPS5 gene and yields better resolution on the intra-specific diversity of the *Babesia* and *Theileria* species. However, the combination of RPS5 nuclear DNA and cox3 DNA tree was more advantageous than the combination of a single gene in the phylogeny of the *Babesia* and *Theileria* species. RPS5 gene, as the phylogenetic marker of *Babesia* and *Theileria* species, tends to have intra-specific diversity and considerable inter-specific variation [78].

In this review, the relationship between the structure and function of the RPS5 protein was summarized. As an important ribosomal protein in the 40s subunit, RPS5, particularly its N-terminus, is intimately related to ribosomal translation, intracellular functions, and the development of liver diseases and cancers, implying that RPS5 could be a potential target for therapeutic intervention in liver diseases. Determining the functions corresponding to the structural domains or sites of RPS5 and modeling the interaction l of drug molecules with RPS5 will greatly contribute to the development of new drugs for the treatment of liver diseases and cancers.

## Figures and Tables

**Table 1:** A sequence alignment produced by ClustalX of Homo sapiens, Rattus rattus, Mus musculus, Podocoryna carnea, and Saccharomyces cerevisiae.

	Homologous	Gene Copy	Nucleotides	Amino Acids	Amino Acid Differences	Highly Conserved C-Terminal Structure and Variable N-Terminal Structure
eukaryotic	RPS5	3~6	701	204	A6 A60 A181	More basic amino acids yes
rat	RPS5	single	701	204 (98% identity)	T6 G60 R181	More basic amino acids yes
mouse	RPS5	3~6	701	204		yes
yeast	RPS5	single	908	204 + 69		4truncated variants (lacking 13, 24, 30, and 46 N-terminal amino acids)
Escherichia coli	RPS7		557	204 − 48		no
				30% identity		

## Data Availability

Not applicable.
